# Supplementary material for: Evidence for a finite-momentum Cooper pair in tricolor d-wave superconducting superlattices
Source: Nat Commun. 2024 May 8;15:3861. doi: 10.1038/s41467-024-47875-4 (PMC11078924; doi:10.1038/s41467-024-47875-4)
Supplement: Supplementary file 1 — Supplementary Information [file 41467_2024_47875_MOESM1_ESM.pdf]

Evidence for a finite-momentum Cooper pair in tricolor d-wave superconducting superlattices

T. Asaba[1,*], M. Naritsuka[2], H. Asaeda[1], Y. Kosuge[1], S. Ikemori[1], S. Suetsugu[1], Y. Kasahara[1], Y. Kohsaka[1], T. Terashima[1], A. Daido[1], Y. Yanase[1], and Y. Matsuda[1,*]

[1]*Department of Physics, Kyoto University, Kyoto 606-8502 Japan and*

[2]*RIKEN Center for Emergent Matter Science, Wako, Saitama 351-0198, Japan*

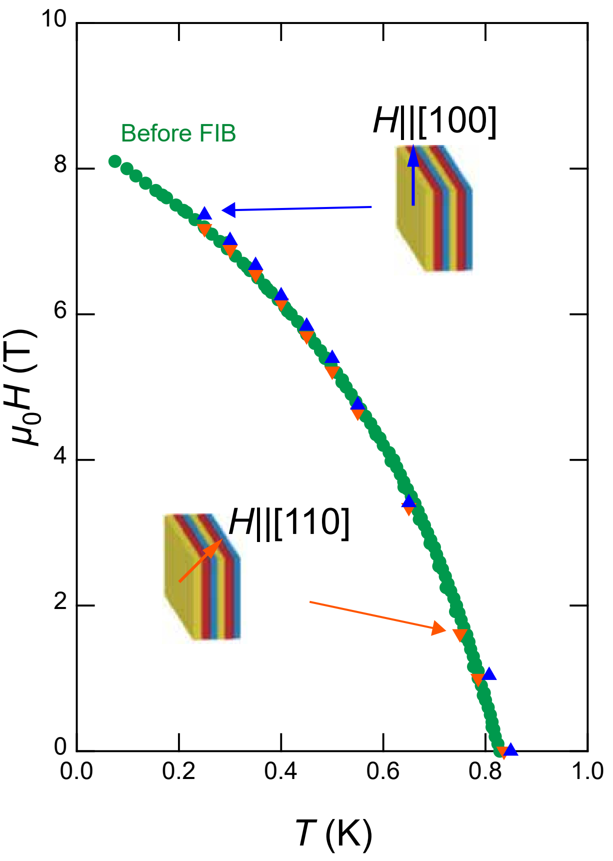

FIG. S1: **Upper critical field H_{c2} for two field directions.** Blue upper-triangles and orange lower-triangles represent H_{c2} from the FIB-cut tricolor superlattice for $\boldsymbol{H} \parallel [100]$ and $\boldsymbol{H} \parallel [110]$, respectively. The current is applied along the [010] direction. For comparison, the H_{c2} curve from the sample before FIB patterning is plotted as green circles.

I. UPPER CRITICAL FIELDS IN PARALLEL FIELDS

Figure S1 shows the upper critical fields in magnetic fields applied parallel to the ab plane plotted as a function of T for two field directions, $\boldsymbol{H} \parallel [100]$ (blue) and $\boldsymbol{H} \parallel [110]$ (orange). Critical fields are determined by using $R_{dc}(T, H) = 0.5R_n(T)$. It is shown that the critical field is almost independent of the field direction, indicating a similar HT-phase diagram for both configurations. The upper critical field before FIB patterning is also shown in green.

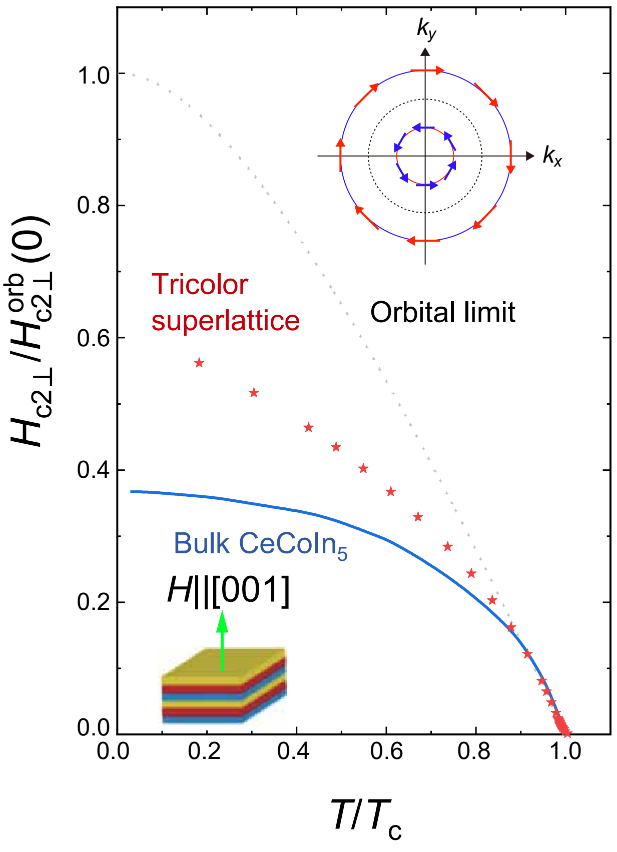

FIG. S2: **Upper critical field $H_{c2\perp}$ for the tricolor lattice and bulk $CeCoIn_5$.** For comparison, the pure orbital limit is also shown in a dotted gray line. The data is taken from Ref. (28). (upper inset) A schematic diagram of Rashba splitting of the Fermi surface with different spin structures.

II. STRONG RASHBA INTERACTION

Direct evidence for the presence of strong Rashba interaction in the present tricolor superlattice is revealed by the upper critical field in magnetic fields applied perpendicular to the ab plane $H_{c2\perp}$. It is well-known that in bulk $CeCoIn_5$ single crystals, the Pauli paramagnetic pair-breaking effect is dominant. The Rashba spin-orbit interaction splits the Fermi surface with spin momentum locking: the spin direction is tilted into the plane, rotating clockwise on one sheet and anticlockwise on the other, as illustrated in the upper inset of Fig. S2. Since the magnetic field applied perpendicular to the 2D plane is orthogonal to the magnetic moment, the Zeeman splitting is absent, leading to the serious reduction of the Pauli paramagnetic effect.

Figure S2 shows $H_{c2\perp}$ normalized by the orbital limiting field $H_{c2\perp}^{orb}(0)$ plotted as a function of normalized temperature T/T_c. Here $H_{c2\perp}^{orb}(0)$ is the zero-temperature value of the orbital upper critical field, which is determined from the initial slope of $H_{c2\perp}(T)$ at T_c by using Werthamer, Helfand, and Hohenberg (WHH) formula, $H_{c2\perp}^{orb}(0) = -0.73T_c(dH_{c2\perp}/dT)_{T_c}$. For comparison, we also include two extreme cases: $H_{c2\perp}/H_{c2\perp}^{orb}(0)$ for bulk $CeCoIn_5$, where $H_{c2\perp}$ is dominated by Pauli pair-breaking effect and the WHH curve with no Pauli effect. Remarkably, $H_{c2\perp}/H_{c2\perp}^{orb}(0)$ of the tricolor superlattice is dramatically enhanced from that of the bulk $CeCoIn_5$ and approaches to the WHH curve with no Pauli effect. This enhancement of $H_{c2\perp}/H_{c2\perp}^{orb}(0)$ is attributed to the increase of the relative importance of the orbital pair-breaking effect compared to the Pauli pair-breaking effect, which appears as a consequence of the strong Rashba spin-orbit interaction in the tricolor Kondo superlattice.

III. NON-RECIPROCAL TRANSPORT MEASUREMENTS WITH THE CURRENT APPLIED 45° AWAY FROM AND PARALLEL TO THE MAGNETIC FIELD

In Fig. S3, the field dependence of the non-reciprocal transport for three configurations, (1)$\boldsymbol{H} \parallel [110]$ and $\boldsymbol{I} \parallel [100]$ (top), (2)$\boldsymbol{H} \parallel [100]$ and $\boldsymbol{I} \parallel [110]$ (middle), and (3) $\boldsymbol{H} \parallel \boldsymbol{I} \parallel [100]$ (bottom) at $T = 300$ mK. The high-field dip anomaly is observed at around 7 T in (1), but it is absent in (2). Together with the results shown in Fig. 3, the presence/absence of the dip anomaly depends on the field direction but not on the current direction. For (3), no non-reciprocal response is observed.

IV. NON-RECIPROCAL TRANSPORT MEASUREMENTS WITH A TILT ANGLE

In Fig. S4, the field dependence of the non-reciprocal transport with magnetic fields applied slightly away from a-axis (a tilt angle $\theta = 4.2°$) is shown. For comparison, the data with magnetic fields applied exactly in-plane is also shown. For both cases, $\boldsymbol{I} \parallel [100]$ and the magnetic field is applied perpendicular to the current ($\boldsymbol{H} \parallel [010]$ for $\theta = 0$), and $T = 250$ mK. The high-field dip anomalies are observed in both cases.

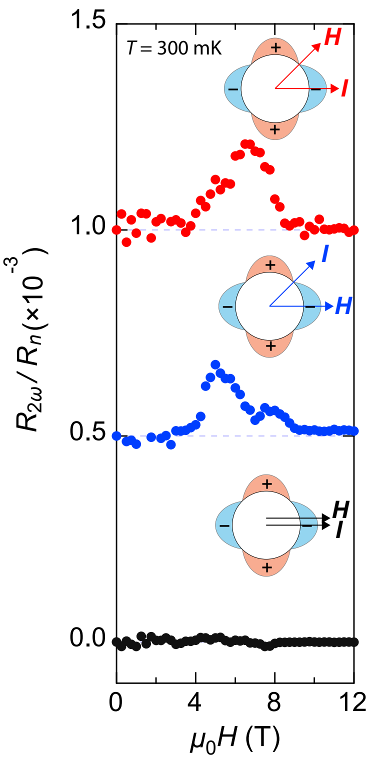

FIG. S3: **Non-reciprocal transport measurements with the field applied 45° away from the current.** (top) $\boldsymbol{H} \parallel [110]$ and $\boldsymbol{I} \parallel [100]$. (middle) $\boldsymbol{H} \parallel [100]$ and $\boldsymbol{I} \parallel [110]$. (bottom) $\boldsymbol{H} \parallel \boldsymbol{I} \parallel [100]$. The curves are vertically shifted for clarity.

V. H_{c2} WITH DIFFERENT CRITERIA

As shown in Fig. S5, H_{c2} was alternatively defined at a resistance of 0.1 R_n (dashed line) in addition to 0.5 R_n (solid line). This highlights that the intrinsic contribution of NRET predominantly becomes discernible in the resistance range between $R = 0.1R_n$ and $R = 0.5R_n$.

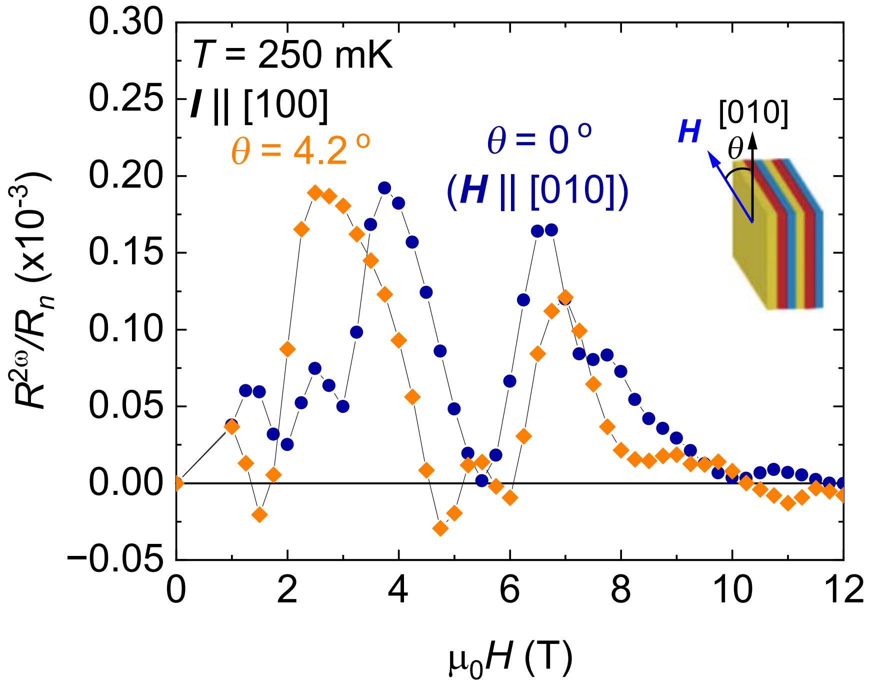

FIG. S4: **Non-reciprocal transport measurements with the field applied 4.2° away from the plane.** For comparison, the data with the field applied exactly in-plane is also shown. The black line indicates the base line.

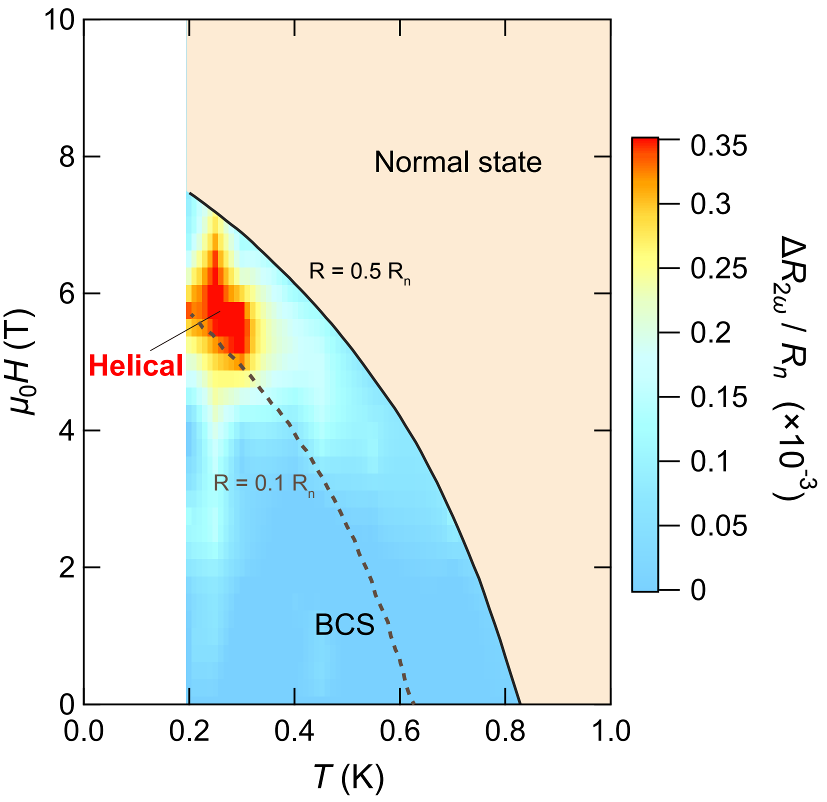

FIG. S5: **A phase diagram with two H_{c2} criteria.** Solid and dashed lines represent $R = 0.5R_n$ and $R = 0.1R_n$, respectively.
